# Exploring Sexual Dimorphism and Asymmetry in Quail (*Coturnix coturnix*) Feet Using Geometric Morphometrics

**DOI:** 10.3390/vetsci12090871

**Published:** 2025-09-08

**Authors:** Barış Can Güzel, Burak Ünal, Mehmet Eroğlu, Fatma İşbilir, Tomasz Szara

**Affiliations:** 1 Department of Anatomy, Faculty of Veterinary Medicine, Siirt University, 56100 Siirt, Türkiye; baris.guzel@siirt.edu.tr (B.C.G.); fatma.isbilir@siirt.edu.tr (F.İ.); 2 Department of Anatomy, Faculty of Veterinary Medicine, Istanbul University-Cerrahpasa, 34320 Istanbul, Türkiye; burak.unal@iuc.edu.tr; 3 Department of Animal Science, Faculty of Veterinary Medicine, Siirt University, 56100 Siirt, Türkiye; mehmet.eroglu@siirt.edu.tr; 4 Department of Morphological Sciences, Institute of Veterinary Medicine, Warsaw University of Life Sciences-SGGW, 02-776 Warsaw, Poland

**Keywords:** avian morphology, bilateral asymmetry, foot shape variation, developmental stability, locomotor adaptation

## Abstract

Understanding morphological variation and asymmetry in avian limbs provides essential insights into functional anatomy, locomotor behavior, and developmental stability. In this study, we investigated shape and size variation in the feet of quails (*Coturnix coturnix*) using two-dimensional geometric morphometric methods. A total of 233 animals were analyzed, representing both the left and right feet of male and female individuals. Nine homologous fixed landmarks were digitized on each foot, and configurations were subjected to Generalized Procrustes Analysis, followed by mirroring of right-side landmarks to ensure consistent orientation. Statistical analyses revealed no significant sexual dimorphism in either foot shape or centroid size. Principal Component Analysis indicated that the main shape variation was distributed individually rather than by sex and primarily affected the relative positions of toes and claws. Procrustes ANOVA confirmed that differences between sexes were not greater than expected by chance. Directional and fluctuating asymmetry were evaluated using a bilateral symmetry model to assess bilateral asymmetry. Directional asymmetry indicated consistent left–right differences, while fluctuating asymmetry reflected individual-level developmental instability and comprised the main source of variation. These findings provide a detailed morphological baseline for quail foot structure and highlight the importance of considering asymmetry in studies of avian functional morphology. The approach may also be a reference for future research into developmental stress, locomotor adaptation, or species-specific anatomical patterns.

## Simple Summary

This study examined the foot morphology of male and female quails using geometric morphometric methods. Two hundred thirty-three animals were analyzed to explore shape, size, and asymmetry differences. Our results showed no significant sexual dimorphism in foot shape or size, suggesting similar ecological and functional roles for both sexes. However, apparent directional asymmetry was found, with one side consistently differing from the other, alongside fluctuating asymmetry, reflecting individual-level developmental variation. These findings contribute to understanding avian limb development and function and highlight the importance of considering asymmetry in morphological and ecological studies of birds.

## 1. Introduction

Sexual dimorphism, a key focus in avian morphology, often arises from sexual selection, habitat differentiation, or functional demands [1]. Morphological differences between sexes may involve subtle shape variations beyond obvious size disparities, offering insights into ecological roles and developmental processes [2]. In monomorphic bird species, external sexual differences are often minimal, and traditional linear measurements may miss subtle variations. Geometric morphometrics (GM), using coordinates of homologous landmarks, enables precise assessment of shape variation [3,4]. Compared to classical methods, GM provides statistically robust evaluations of micromorphological differences in avian structures [5].

Allometry, the influence of size on shape, is critical in GM studies [6]. In birds, shape changes due to size may stem from age, sex, or environmental factors [7]. Controlling for allometric effects is essential to avoid misinterpreting size-driven differences as shape variation, particularly in functionally significant structures like feet, where shape changes reflect growth and development [8]. Geometric morphometrics has proven effective in avian research, detecting subtle shape variations linked to function and behavior [2,9]. For instance, partridge species show significant foot shape variation related to sex and species, particularly in toe angles and asymmetry [10].

Additionally, GM is vital for evaluating symmetry and asymmetry [11]. Fluctuating asymmetry (FA), random deviations in bilateral structures, indicates developmental instability due to environmental or genetic stressors [12,13]. Assessing FA in functionally important organs like bird feet can reveal ecological and evolutionary patterns. Although *Coturnix coturnix* is a common model in anatomical research, detailed studies on foot morphology and asymmetry are limited. This study uses two-dimensional GM to quantify shape and size variation in quail feet, characterize asymmetry patterns, and evaluate their alignment with functional demands or developmental noise, contributing to understanding avian limb morphology.

Understanding the form and function of avian feet is crucial for interpreting their ecological roles, locomotor strategies, and evolutionary adaptations; although *Coturnix coturnix* is a widely used model in developmental, behavioral, and anatomical research, detailed investigations into the morphological variation and bilateral asymmetry of its foot structure remain scarce, despite the feet being the primary interface with the environment and playing a key role in balance, movement, and substrate interaction—where even subtle shape differences may reflect underlying biological, functional, or developmental processes, including directional (DA) or fluctuating (FA) asymmetry, which can offer insights into developmental stability, locomotor preference, and sexual dimorphism; thus, by applying two-dimensional geometric morphometric techniques, this study aims to quantify shape and size variation in male and female quail feet, detect and characterize asymmetry patterns, and evaluate whether these patterns align with functional demands or result from developmental noise, thereby contributing to a deeper understanding of avian limb morphology from both functional and evolutionary perspectives.

## 2. Materials and Methods

### 2.1. Ethical Statement

The research material was obtained from a slaughterhouse. No animals were harmed for this study. Since slaughterhouse materials are not subject to ethics committee permission, an ethics committee decision was not required for this study.

### 2.2. Samples

A total of 233 quails (116 females and 117 males) were analyzed, resulting in 466 images (left and right feet of each individual). All individuals were sexually mature adults, approximately 6–8 weeks old, and were obtained from a commercial slaughterhouse located in Siirt, Türkiye, where birds are processed at a standardized post-pubertal age under uniform rearing conditions. Birds with visible deformities or pathological abnormalities were excluded from the study.

### 2.3. Sample Preparation

**Figure 1:**
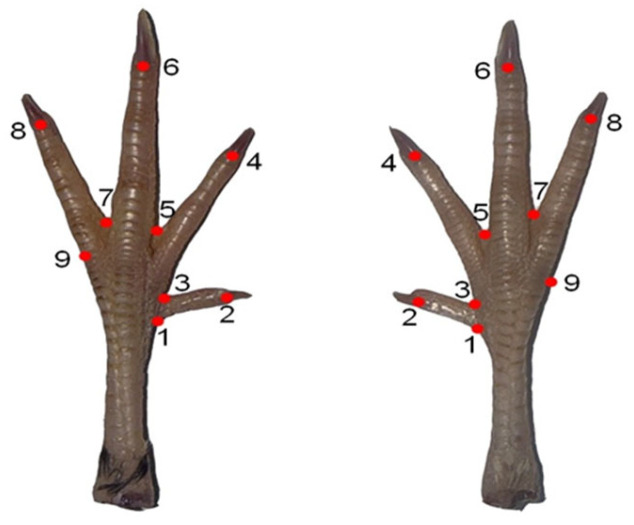
Nine homologous landmarks digitized on the feet of quails (*Coturnix coturnix*) for two-dimensional geometric morphometric analysis: Lm1: Lateral point of the articulatio metatarsophalangea I. Lm2: Phalanx terminalis digiti I. Lm3: Medial point of the articulatio metatarsophalangea I. Lm4: Phalanx terminalis digiti II. Lm5: Tela interdigitalis intermedia. Lm6: Phalanx terminalis digiti III. Lm7: Tela interdigitalis lateralis. Lm8: Phalanx terminalis digiti IV. Lm9: Lateral point of the articulatio metatarsophalangea IV.

The images were converted into Tps format using TpsUtil software (version 1.74) for the analyses. Subsequently, landmarks were placed on the Tps-formatted photographs. Coordinates of each landmark were digitized using tpsDig software (version 2.30). A total of nine landmarks were used in the analysis (Figure 1).

### 2.4. Statistical Analyses

Four hundred and sixty-six quail foot images were used for shape analysis, comprising both left and right feet from male and female individuals. Nine homologous fixed landmarks were digitized on each image using a two-dimensional TPS file format. Landmark data were imported into R (version 4.4.2) and processed using the geomorph package (version 4.0.10).

All landmark configurations were subjected to a Generalized Procrustes Analysis (GPA) to remove variation due to translation, rotation, and scale. Since left and right feet were included, mirror reflection of right foot landmarks was performed before shape analyses to standardize orientation.

Principal Component Analysis (PCA) was performed on Procrustes-aligned coordinates to assess shape variation. The first few principal components (PCs) explaining more than 10% of the total variation were examined in detail.

For group comparisons, a Procrustes ANOVA (via procD.lm) was used to test the effects of sex, side (left vs. right), and their interaction on shape. The significance of directional asymmetry (DA), fluctuating asymmetry (FA), and individual variation was tested using the bilateral asymmetry function with 999 permutations.

Centroid size (CS) was calculated as the square root of the sum of squared distances from each landmark to the centroid of the landmark configuration, extracted following Generalized Procrustes Analysis (GPA). As GPA standardizes landmark configurations by removing variation due to translation, rotation, and scale, CS is a unitless measure representing the overall size of the foot configuration. CS values were used as a proxy for foot size in comparisons between sexes and sides. Differences in CS between sexes were evaluated using Welch’s *t*-test and a permutation-based ANOVA to assess potential sexual size dimorphism.

All permutation-based tests used 999 iterations, and effect sizes were interpreted based on F-values, R^2^, and Z-scores.

## 3. Results

### 3.1. Principal Component Analysis of Reflected Quail Foot Shapes

**Figure 2:**
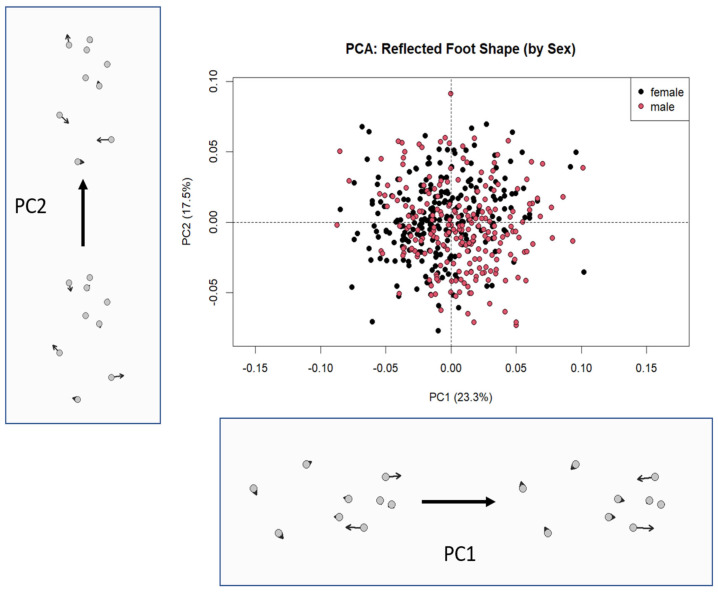
Principal Component Analysis (PCA) of Reflected Quail Foot Shapes with Associated Shape Deformations along PC1 and PC2 Axes. The central scatterplot displays the distribution of individuals (*n* = 466) based on the first two principal components (PC1: 23.3%; PC2: 17.5%) of shape variation after reflection of right-side landmarks. Male and female individuals are color-coded. The deformation grids illustrate the shape differences corresponding to the negative and positive extremes of PC1 (bottom) and PC2 (left), with arrows representing landmark displacement from the mean shape.

Following the reflection of right foot landmarks to eliminate directional asymmetry, a Principal Component Analysis (PCA) was performed on the Procrustes-aligned shape coordinates of 466 quail feet. The analysis revealed that shape variation was distributed across several principal components, with the first three explaining most of the total variation (Figure 2).

The first principal component (PC1) accounted for 23.3% of the total shape variance and primarily described changes in the mediolateral spread of the toes. Individuals with positive PC1 scores exhibited a wider separation between the anterior digits, particularly between landmarks corresponding to the outer toe tips (landmarks 4 and 8). Conversely, individuals with negative PC1 scores showed a more clustered toe arrangement, suggesting reduced toe spreading.

The second principal component (PC2), explaining 17.5% of the variance, represented variation in the vertical or proximodistal extension of the toes. As PC2 values increased, the anterior digits (especially landmark 6) projected further forward, indicating longer or more extended toes. Negative PC2 scores were associated with more retracted or contracted toe positions.

The PCA scatterplot showed a broad but overlapping distribution of male and female individuals, without a clear separation between sexes. Although shape variation was observed among individuals, there was no clear sexual differentiation in morphospace, suggesting that sexual dimorphism in foot shape is minimal.

### 3.2. Size and Shape (Centroid Size) Comparison

To assess sexual size dimorphism in quail foot morphology, centroid size (CS) was compared between male and female individuals (n = 466). The average CS was 1.0899 ± 0.0096 for females (n = 235) and 1.0888 ± 0.0098 for males (n = 231). An independent samples t-test revealed no statistically significant difference in centroid size between sexes (t = 1.23, *p* = 0.221).

This result was confirmed through a permutation-based ANOVA using 1000 iterations (F = 1.505, *p* = 0.224, Rsq = 0.0032), indicating that foot size is not significantly sexually dimorphic in this dataset.

A Procrustes ANOVA was conducted using reflected and aligned shape coordinates to evaluate shape differences between sexes. The results showed a non-significant effect of sex on overall foot shape. Although individual variation is evident, the between-group (sex) variation does not exceed what would be expected by chance alone under the null hypothesis.

These results collectively suggest that male and female quails exhibit highly similar foot shapes and sizes, and any minor shape variations are more likely attributable to individual-level differences rather than sexual dimorphism.

### 3.3. Directional Asymmetry and Fluctuating Asymmetry

Significant directional asymmetry was observed in the shape and size of quail feet. The side factor was highly important in the Procrustes ANOVA for shape (F = 23.28, *p* = 0.001, Z = 6.94), explaining approximately 4.9% of total shape variation. Similarly, size-based DA analysis showed that foot size significantly differed between the left and right sides (F = 43.17, *p* = 0.001, Z = 4.40), contributing to 8.5% of the total size variation. These results suggest a consistent left–right difference across individuals, indicating strong directional asymmetry in foot shape and size (Table 1).

The interaction term (Individual × Side) in both shape and size ANOVAs was the most significant contributor to variation (shape Rsq = 72.3%; size Rsq = 68.0%), indicating substantial fluctuating asymmetry (FA). This means that, beyond the consistent left–right difference, individual quails exhibit varying degrees of asymmetry in foot morphology, possibly due to developmental instability or environmental influences.

The individual effect was non-significant in either shape or size, with R-squared values around 23% and *p*-values well above 0.05. This suggests that the main variation between individuals is primarily modulated through asymmetry (FA and DA) rather than outright individual shape or size differences.

## 4. Discussion

This study provides a comprehensive morphometric assessment of foot morphology in *Coturnix coturnix*, emphasizing sexual dimorphism and bilateral asymmetry, using two-dimensional geometric morphometric techniques. The findings offer novel insights into pedal variation’s developmental, functional, and evolutionary implications in this ground-dwelling avian species, with broader relevance for poultry production and behavioral research.

One of the primary findings of the present study is the absence of statistically significant sexual dimorphism in both the size and shape of the foot. This result aligns with the observations of limited sexual dimorphism in *Alectoris chukar* and *Perdix perdix*, with differences primarily restricted to toe angles, possibly reflecting behavioral rather than structural divergence [14]. Similarly, Demircioğlu et al. found non-significant differences in digit ratios between sexes in *Coturnix japonica*, suggesting that functional foot morphology in quails is shaped more by locomotor demands than by sex-based roles [15].

The absence of significant sexual dimorphism in quail foot morphology found in this study aligns with broader patterns observed in other avian taxa, where functional demands and ecological pressures often override sex-based morphological divergence. For example, Gray and Renner [16] similarly reported no significant shape differences in the claws and bills of male and female kākāpō (*Strigops habroptilus*), a species with notable behavioral dimorphism, suggesting that shared ecological roles may suppress morphological differentiation despite strong sexual selection pressures. Additionally, the evolutionary framework of the avian foot, as discussed by Carril et al. [17], emphasizes a trend of structural simplification and conserved modularity across bird lineages, regardless of locomotor specialization. Their anatomical network analysis revealed that although bird feet exhibit considerable morphological diversity, especially at lower taxonomic levels, the underlying musculoskeletal architecture remains highly constrained by evolutionary history, supporting the high degree of individual-level but not sex-based variation observed in our sample. Furthermore, Simons [18] demonstrated in pelecaniform birds that while limb morphology varies with flight mode, most skeletal elements scale isometrically, reinforcing the idea that functional adaptation often occurs within strict morphological limits. In monogamous or low-polygynous species such as quail, reduced male–male competition results in weaker selective pressures for morphological differentiation between sexes [19]. Both sexes engage in comparable terrestrial foraging strategies and habitat use, likely constraining the divergence of foot traits. In this context, the directional and fluctuating asymmetries detected in our study may reflect subtle behavioral laterality or developmental instability, rather than adaptive sexual differentiation. Collectively, these comparative findings highlight the importance of considering phylogenetic constraint, ecological equivalence, and network-level integration when interpreting morphometric variation in avian appendages.

PCA reinforced this interpretation by showing extensive morphospace overlap between sexes. The leading shape components (PC1 and PC2) were associated with toe spread and anterior–posterior elongation, respectively. These variations may reflect individual differences in posture, mechanical loading, or substrate interaction during growth and locomotion. Similar functional implications were reported by Demircioğlu et al., where lateral toe angles were found to be greater than medial angles—an adaptation that may enhance lateral stability during movement [15].

Notably, the study also revealed significant bilateral asymmetry in foot morphology. Directional asymmetry (DA) was consistently observed, with the right foot being larger and extended, which may suggest functional lateralization. This finding is consistent with studies in birds demonstrating limb dominance and lateralized behaviors—such as preferential foot use during locomotion or prey handling—as underlying causes of consistent morphological asymmetry [20,21]. For instance, Rogers and Workman [22] demonstrated that asymmetrical hatching behaviors in domestic chicks (*Gallus gallus*) influence the development of postnatal laterality, suggesting that early behavioral biases can contribute to morphological asymmetries in avian limbs. Such asymmetries may develop from repetitive, lateralized behaviors that exert asymmetric biomechanical loads on developing limbs.

In addition to DA, a high fluctuating asymmetry (FA) level was detected, accounting for more than 70% of the total shape variation. FA represents random deviations from perfect bilateral symmetry and is commonly interpreted as an indicator of developmental instability [23]. Elevated FA levels may point to underlying stressors during ontogeny, such as nutritional deficiencies, environmental fluctuations, or genetic variability [24]. The consistent presence of high FA across individuals suggests that even subtle developmental perturbations can significantly influence morphological outcomes in avian feet.

Although directional asymmetry accounted for a relatively small proportion of total variation (4.9% for shape and 8.5% for size) compared to fluctuating asymmetry (FA, ~72%), this pattern is consistent with findings from previous morphometric studies, where DA typically explains a smaller share of variance than FA due to the random nature of FA dominating overall variability [23,25]. Importantly, despite its lower contribution, DA was highly significant for both shape and size (shape: F = 23.28, Z = 6.94, *p* = 0.001; size: F = 43.17, Z = 4.40, *p* = 0.001), indicating that the left–right differences represent a consistent, biologically meaningful asymmetry rather than statistical noise. This suggests that, while individual developmental instability (FA) predominates in quail foot morphology, the presence of a robust DA component may reflect functional or behavioral lateralization in this species.

### Evolutionary Implications

From an evolutionary perspective, the patterns observed in this study echo broader trends in avian and non-avian theropod evolution. Hutchinson and Allen emphasized a functional continuum in limb evolution from theropod dinosaurs to modern birds, highlighting shifts from hip-driven to knee-driven locomotion and the incorporation of the hallux into weight-bearing structures [26]. These transitions may have facilitated the diversification of avian pedal morphologies and laid the groundwork for functional adaptations and individual variation in foot structure. Paleontological data further support this connection. For instance, McIntosh demonstrated that in the early bird *Confuciusornis sanctus*, claw morphology and foot structure were tightly linked to behavioral and ecological functions, indicating that pedal specializations evolved early in avian history. The asymmetries and individual variation observed in quails may thus reflect an inherited evolutionary flexibility in foot development [27].

Comparative studies reinforce the ecological basis of foot morphology variation. Abourachid et al. showed that arboreal birds adopt distinct morphologies to achieve similar grasping functions [28], whereas ground-dwelling species exhibit more stability-oriented features. Similarly, Middleton demonstrated that the orientation and reversal of the hallux influence grasping capability and balance, affecting overall foot mechanics [29]. Although quails are not arboreal, such findings support the notion that ecological pressures significantly influence foot design. Recent three-dimensional morphometric studies have further clarified this ecological–morphological relationship. De Mendoza et al. reported that the shape of the tarsometatarsus in *Anseriformes* is strongly associated with habitat use [30]. Segesdi et al. [31] found that aquatic birds exhibit specific limb morphologies aligned with their locomotor strategies. Though *Coturnix coturnix* is terrestrial, these studies highlight how ecological demands rather than sexual dimorphism are primary drivers of limb variation. Moreover, Carril et al. [32] used anatomical network analysis to demonstrate that the underlying musculoskeletal modularity remains highly conserved despite a wide range of pedal morphologies among birds. These findings suggest that despite external variation, avian pedal morphology is constrained by conserved musculoskeletal structures, maintained by developmental constraints and stabilizing selection.

Finally, the link between pedal structure and ecological function has also been demonstrated by Hedrick et al. [33], who showed that claw curvature and phalangeal proportions vary predictably with behavioral ecology. While quails lack extreme adaptations for climbing or predation, the subtle asymmetries and individual variation found in this study may reflect fine-scale responses to specific locomotor or foraging demands within complex terrestrial environments. Notably, the use of geometric morphometrics in this study provided a sensitive and robust framework for detecting shape differences that might be overlooked by traditional morphometrics. Landmark-based methods enable detailed quantification of form, spatial relationships, and asymmetry in biological structures [34], making them particularly suitable for assessing the subtle yet biologically meaningful variation observed in quail feet.

## 5. Conclusions

In conclusion, the findings of this study demonstrate that while male and female quails show highly similar foot morphologies, directional and fluctuating asymmetries are prominent and biologically significant. These results offer new insights into the species’ functional morphology, developmental biology, and ecological adaptations. The asymmetry patterns observed here may reflect a combination of behavioral laterality, environmental influences, and developmental variability. Further studies incorporating environmental data, behavioral observations, or ontogenetic comparisons would deepen our understanding of the underlying causes and consequences of asymmetry in avian morphology.

## Figures and Tables

**Table 1:** Procrustes ANOVA Results for Directional and Fluctuating Asymmetry in Quail (Coturnix coturnix) Feet. The table summarizes the effects of individual, side (directional asymmetry, DA), and their interaction (fluctuating asymmetry, FA) on foot shape and centroid size, based on 466 foot images. Columns include degrees of freedom (Df), sum of squares (SS), mean squares (MS), explained variance (Rsq), F-statistic (F), Z-score (Z), and *p*-value. Significant DA (*p* ≤ 0.001) indicates consistent left–right differences, while high FA (Rsq = 72.3% for shape, 68.0% for size) reflects individual-level developmental variation.

Trait	Source	Df	SS	MS	Rsq	F	Z	*p*-Value
Shape	Individual	119	0.50312	0.00423	0.228	0.916	−1.615	0.951
Side (DA)	1	0.10741	0.10741	0.049	23.28	6.940	0.001	***
Ind × Side (FA)	345	1.59201	0.00462	0.723	—	—	—	
Centroid Size	Individual	119	0.01029	0.000086	0.234	0.999	−0.022	0.508
Side (DA)	1	0.00374	0.00374	0.085	43.17	4.404	0.001	***
Ind × Side (FA)	345	0.02986	0.000087	0.680	—	—	—	

Rsq: Explained variance; DA: Directional asymmetry; FA: Fluctuating asymmetry; *** *p* ≤ 0.001.

## Data Availability

The data presented in this study are available upon request from the corresponding author (T.S.). The data are not publicly available due to them being part of an ongoing research study.
